# Reassessment of the evolution of wheat chromosomes 4A, 5A, and 7B

**DOI:** 10.1007/s00122-018-3165-8

**Published:** 2018-08-23

**Authors:** Jan Dvorak, Le Wang, Tingting Zhu, Chad M. Jorgensen, Ming-Cheng Luo, Karin R. Deal, Yong Q. Gu, Bikram S. Gill, Assaf Distelfeld, Katrien M. Devos, Peng Qi, Patrick E. McGuire

**Affiliations:** 1 Department of Plant Sciences, University of California, Davis, CA USA; 2 Crop Improvement and Genetics Research, USDA-ARS, Albany, CA USA; 3 Department of Plant Pathology, Kansas State University, Manhattan, KS USA; 4 School of Plant Sciences and Food Security, Tel Aviv University, Tel Aviv, Israel; 5 Institute of Plant Breeding, Genetics and Genomics, Department of Crop and Soil Sciences, University of Georgia, Athens, GA USA; 6 Department of Plant Biology, University of Georgia, Athens, GA USA

## Abstract

**Key message:**

Comparison of genome sequences of wild emmer wheat and *Aegilops tauschii* suggests a novel scenario of the evolution of rearranged wheat chromosomes 4A, 5A, and 7B.

**Abstract:**

Past research suggested that wheat chromosome 4A was subjected to a reciprocal translocation T(4AL;5AL)1 that occurred in the diploid progenitor of the wheat A subgenome and to three major rearrangements that occurred in polyploid wheat: pericentric inversion Inv(4AS;4AL)1, paracentric inversion Inv(4AL;4AL)1, and reciprocal translocation T(4AL;7BS)1. Gene collinearity along the pseudomolecules of tetraploid wild emmer wheat (*Triticum turgidum* ssp. *dicoccoides,* subgenomes AABB) and diploid *Aegilops tauschii* (genomes DD) was employed to confirm these rearrangements and to analyze the breakpoints. The exchange of distal regions of chromosome arms 4AS and 4AL due to pericentric inversion Inv(4AS;4AL)1 was detected, and breakpoints were validated with an optical Bionano genome map. Both breakpoints contained satellite DNA. The breakpoints of reciprocal translocation T(4AL;7BS)1 were also found. However, the breakpoints that generated paracentric inversion Inv(4AL;4AL)1 appeared to be collocated with the 4AL breakpoints that had produced Inv(4AS;4AL)1 and T(4AL;7BS)1. Inv(4AS;4AL)1, Inv(4AL;4AL)1, and T(4AL;7BS)1 either originated sequentially, and Inv(4AL;4AL)1 was produced by recurrent chromosome breaks at the same breakpoints that generated Inv(4AS;4AL)1 and T(4AL;7BS)1, or Inv(4AS;4AL)1, Inv(4AL;4AL)1, and T(4AL;7BS)1 originated simultaneously. We prefer the latter hypothesis since it makes fewer assumptions about the sequence of events that produced these chromosome rearrangements.

**Electronic supplementary material:**

The online version of this article (10.1007/s00122-018-3165-8) contains supplementary material, which is available to authorized users.

## Introduction

The genome of hexaploid bread wheat (*Triticum aestivum*, 2*n *= 6*x *= 42) consists of three subgenomes designated as A, B, and D. The A subgenome was contributed by wild einkorn wheat *T. urartu* (Dvorak et al. 1988, 1993), the B subgenome was contributed by an unknown species closely related to *Aegilops speltoides* (Dvorak and Zhang 1990), and the D subgenome was contributed by *Ae. tauschii* (Kihara 1944; McFadden and Sears 1946; Wang et al. 2013). Based on the subgenome membership and the ability to genetically compensate for each other in nullisomic-tetrasomic stocks, most of the 21 bread wheat chromosomes could be assigned to a subgenome and one of the seven homoeologous chromosome groups (Sears 1966).

Chromosome 4A was exceptional. At first, the chromosome was erroneously assigned to the B subgenome. The error was detected, and the chromosome was reallocated to the A subgenome (Dvorak 1983; Dvorak et al. 1990). Second, the chromosome did not pair in *T. aestivum* × *T. urartu* hybrids, while each of the remaining six chromosomes of the A subgenome did pair (Chapman et al. 1976; Dvorak 1976). In the absence of expression of the wheat *Ph1* locus, which precludes pairing of homoeologous chromosomes in wheat, bread wheat chromosome 4A paired with the short arms of chromosomes of homoeologous group 7, in addition to chromosomes of homoeologous group 4, while 5A paired with the long arm of group 4 chromosomes, indicating that 4A was involved in reciprocal translocations with chromosome arms 5AL and 7BS (Naranjo et al. 1987).

Subsequent comparative genetic and deletion mapping revealed that the chromosome contained additional rearrangements. The chromosome appeared to be involved in two pericentric inversions and one paracentric inversion in the long arm, in addition to the already mentioned reciprocal translocations with arms 5AL and 7BS (Naranjo et al. 1987; Devos et al. 1995; Mickelson-Young et al. 1995; Nelson et al. 1995; Miftahudin et al. 2004; Ma et al. 2014; Jorgensen et al. 2017). For the sake of clarity and simplicity, we designate throughout this paper the reciprocal translocation of 4A with 5AL as T(4AL;5AL)1, the reciprocal translocation with 7BS as T(4AL;7BS)1, the major pericentric inversion as Inv(4AS;4AL)1, the minor pericentric inversion involving a pericentric region of 4A (Miftahudin et al. 2004; Ma et al. 2014) as Inv(4AS;4AL)2, and the 4AL paracentric inversion as Inv(4AL;4AL)1. In results, we give each rearrangement an explicit name based on an earlier rearrangement-naming proposal (Dvorak et al. 2018).

The reciprocal translocation T(4AL;5AL)1 must have taken place in the diploid ancestor of the wheat A subgenome since it exists in diploid einkorn wheat (*T. monococcum*) (Dubcovsky et al. 1996). The remaining rearrangements took place in polyploid wheat.

These rearrangements created the present-day polyploid wheat chromosome 4A. Based on the proposed rearrangements, the chromosome is expected to consist of syntenic blocks of ancestral chromosomes arranged in the following order starting at the tip of the present-day short arm and neglecting Inv(4AS;4AL)2: 4AS-4AL-centromere-4AS-5AL-4AL-7BS. Deletion mapping of the bread wheat 4A chromosome with expressed sequence tag (EST) markers identified six EST loci that appeared to confirm the breakpoints of Inv(4AL;4AL)1, which inverted 5AL and 4AL segments in the long arm into their present-day position, and two EST loci that appeared to confirm the existence of an ancient 4AS segment at the tip of the present-day arm 4AS (Miftahudin et al. 2004).

A single nucleotide polymorphism (SNP) ultra-dense genetic map based on a mapping population of durum wheat (*T. turgidum* ssp. *durum*, 2*n *= 4*x *= 28, subgenomes AABB) cv ‘Langdon’ (LDN) × wild emmer wheat (*T. turgidum* ssp. *dicoccoides,* 2*n *= 4*x *= 28, subgenomes AABB) accession PI 428082 (henceforth, the LDN × PI 428082 genetic map) was employed to confirm these findings (Jorgensen et al. 2017). Wild emmer wheat is the progenitor of cultivated tetraploid wheat, including durum. Recombination along the entire length of chromosome 4A in this mapping population and another from a durum × wild emmer wheat cross (Avni et al. 2014; Jorgensen et al. 2017) showed that the structure of the wild emmer wheat chromosome 4A and durum wheat chromosome 4A must be the same. The presence of the rearrangements in wild emmer wheat 4A was confirmed by the wild emmer wheat genome sequence (Avni et al. 2017). Analyses of genetic diversity along the bread wheat and wild emmer wheat chromosome 4A suggested that the rearrangements in chromosome 4A originated early in the evolution of wild emmer wheat or were contemporary with its speciation (Jorgensen et al. 2017).

The synteny block translocated into 4AL from 5AL and a synteny block of 4AL translocated into the ancient 4AS from the ancient 4AL by pericentric inversion Inv(4AS;4AL)1 are in an inverted orientation (Devos et al. 1995; Mickelson-Young et al. 1995), which was confirmed by the LDN × PI 428082 genetic map (Jorgensen et al. 2017). However, the genetic map failed to confirm the locations of the six ESTs reported to delineate the breakpoints of the putative inversion Inv(4AS;4AL)1 (Miftahudin et al. 2004). Similar findings were made with a physical map of bread wheat 4A (Hernandez et al. 2012). A radiation hybrid map of 4A revealed discrepancies in the 4A deletion map (Balcárková et al. 2017), which undoubtedly caused the discrepancies between EST deletion mapping on one hand (Miftahudin et al. 2004) and physical and genetic mapping on the other hand (Hernandez et al. 2012; Jorgensen et al. 2017). The LDN × PI 428082 genetic map confirmed the existence of the pericentric inversion Inv(4AS;4AL)2 identified by Miftahudin et al. (2004), but failed to confirm the location of ESTs that were used as evidence for the existence of the ancient 4AS synteny block in the present-day 4AS.

Here, we employ comparisons of the genome sequences of wild emmer wheat accession ‘Zavitan’ (Avni et al. 2017) and *Ae. tauschii* accession AL8/78 (Luo et al. 2017) in delimiting synteny blocks in the wild emmer wheat chromosomes 4A, 5A, and 7B and in the analyses of the breakpoints delimiting these synteny blocks. Sequencing of the large *Triticeae* genomes is at the limit of the current genome sequencing technology, and it is possible that some of the rearrangements detected in a genome sequence may not be real. To prevent mistaking assembly errors for rearrangements, it is prudent to use a sequence-independent representation of the genome to validate rearrangements detected in a genome sequence. We employ for that purpose optical Bionano genome (BNG) maps (Hastie et al. 2013; Dvorak et al. 2018). A BNG map consists of contigs assembled from overlaps among high-molecular-weight DNA molecules digested with a single-strand restriction endonuclease (nickase). The nicks are labeled with fluorescent nucleotides, and distances between them are optically measured on DNA molecules aligned and stretched in nano-channels (Xiao et al. 2007). If a BNG map and genome sequence show the same distribution of nickase restriction sites, the sequence is validated. A BNG map of wild emmer accession Zavitan (Dvorak et al. 2018) was used to validate breakpoints of inversions and translocations detected in the wild emmer wheat genome sequence. BNG maps of four different *Ae. tauschii* accessions (Luo et al. 2017; Dvorak et al. 2018), including AL8/78, were used to validate the regions of the *Ae. tauschii* genome sequence corresponding to the breakpoints in the wild emmer wheat pseudomolecules.

In a previous comparison (Dvorak et al. 2018), 38,775 high confidence (HC) genes annotated in the *Ae. tauschii* pseudomolecules (Luo et al. 2017) were used as queries in BLASTP searches against the genes annotated in the A and B subgenomes of wild emmer wheat (Avni et al. 2017). Here, we generated a reciprocal comparison, in which 4350 genes annotated in the wild emmer pseudomolecule 4A (Avni et al. 2017) were used as queries in BLASTP searches against the 38,775 HC genes annotated in the *Ae. tauschii* pseudomolecules. The best BLASTP hits were arranged in a spreadsheet matrix in both comparisons. The *Ae. tauschii* pseudomolecules were used as representations of the ancestral state of wheat chromosomes 4, 5, and 7. The order of the best BLASTP hits in the wild emmer wheat, and *Ae. tauschii* pseudomolecules was used to reconstruct homoeology between *Ae. tauschii* chromosomes 4D, 5D, and 7D and wild emmer wheat chromosomes 4A, 5A, and 7B, to infer the boundaries of synteny blocks in these chromosomes, and to detect rearrangement breakpoints. These analyses confirmed conclusions made with the LDN × PI 428082 genetic map and led us to propose a new scenario of evolution of wheat chromosomes 4A and 7B.

## Materials and methods

### Gene collinearity and structural chromosome analyses

The methodology used for gene collinearity analysis has been described earlier (Luo et al. 2017), and only information specific for this study will be provided here. The amino acid sequences of 4350 HC genes located on the wild emmer wheat pseudomolecule 4A were downloaded from Avni et al. (Avni et al. 2017), and those of the *Ae. tauschii* 38,775 HC genes located on the *Ae. tauschii* pseudomolecules (Luo et al. 2017) were downloaded from (http://aegilops.wheat.ucdavis.edu/ATGSP/annotation/). BLASTP homology searches using amino acid sequences of the wild emmer wheat HC genes as queries and amino acid sequences of the *Ae. tauschii* genes as targets were performed. A default BLASTP parameter setting was used. The top three alignment scores were recorded, ranked, and the top hits were sorted by the position of genes on the 4A pseudomolecules in ascending order.

Collinearity of top hits in the *Ae. tauschii* genome (target) was assessed as follows. A spreadsheet matrix was constructed, which consisted of wild emmer query genes ordered vertically in ascending order starting with the short arm terminus at the top (Online Resource 1). The coordinates of the top hits in the *Ae. tauschii* genome were placed into the cells at the intersections of the query gene rows and the relevant pseudomolecule columns. Three or more genes were considered collinear if the starting nucleotides of the top hits followed an ascending or descending order and distances between them were < 5 Mb on the *Ae. tauschii* pseudomolecule. Noncollinear genes interrupting a sequence of collinear genes were allowed. If a wild emmer wheat gene was homologous to a duplicated gene on a target pseudomolecule, only one of the duplicated genes was considered as collinear. The cells including target genes collinear with the query genes were color-coded; cells containing coordinates of noncollinear genes were left colorless. Changes in gene order due to inversions or translocations were indicated by changes in cell color. The ancestral order of collinear genes was light green. Inverted order was dark green or any other color if several inversions were in the same region. Cells containing collinear genes of intrachromosomal translocations were blue.

### Naming rearrangements

The following rules were suggested for naming rearrangements using the rearrangement database for Poaceae reported earlier (Dvorak et al. 2018). Inversions were abbreviated as Inv and translocations as T. The abbreviation was followed by the genome(s) in which the rearrangement was detected, A for the wheat A subgenome, B for the wheat B subgenome, Aet for the *Ae. tauschii* genome, etc. The location of a rearrangement was defined by the names of HC genes in gene set v2.0 annotated in the *Ae. tauschii* genome assembly v4.0. Thus, e.g., TA(AET4Gv20728400-30000) is a translocated synteny block observed in the A subgenome and homoeologous to *Ae. tauschii* pseudomolecule 4D (AET4). The region starts with *Ae. tauschii* gene AET4Gv20728400 and ends with gene AET4Gv20730000. Naming of reciprocal translocations and pericentric inversions is more complicated and requires defining the breakpoints. Thus, e.g., TA(AET4Gv20754000:5100;AET5Gv21126000:200) is a translocation in the A subgenome involving a breakpoint in chromosome 4A between genes collinear with *Ae. tauschii* 4D genes AET4Gv20754000 and AET4Gv2075100 and a breakpoint in chromosome 5A between genes collinear with *Ae. tauschii* 5D genes AET5Gv21126000 and AET5Gv21126200. The *Ae. tauschii* pseudomolecule coordinate (gene start) for each of the 38,775 *Ae. tauschii* HC genes is given in Online Resource 1 (Dvorak et al. 2018).

### Dot-plots and satellite DNA search

Annotated primary transcripts and corresponding protein sequences in the emmer wheat Zavitan assembly and the *Ae. tauschii* AL8/78 assembly v4.0 were downloaded. Only the first transcript for each gene was retrieved. A BLASTP search was conducted using the *Ae. tauschii* proteins as queries and the emmer wheat proteins as targets. The top two hits with an *E* value < 1e−5 were recorded. Homologous gene pairs identified by BLASTP were used to detect syntenic blocks using the software MCscanX (Wang et al. 2012). Collinear segments for all possible pairs of chromosomes were detected using a match score of 50, a gap penalty of − 1, an *E* value threshold of 1e−05, a minimum of three genes, and maximum gap size of 25 between two consecutive proteins to declare a collinear block. Pairwise comparative dot-plots using the MCscanX output were drawn using R.

To search for satellite DNA in breakpoint regions, nucleotide sequences were extracted from the wild emmer wheat pseudomolecules based on the coordinates for the breakpoint using the iTools Fatools “extract” module (He et al. 2013). Each extracted sequence was aligned against itself with YASS (Noe and Kucherov 2005) using scoring matrix match 5, transversion − 4, transition − 3, other − 4; gap open − 16, gap extension − 4. The dot-plot was generated with YASS. Tandem repeats were detected in each sequence with tandem repeats finder (TRF) (Benson 1999) using match, mismatch, delta, PM, PI, minscore, and maxperiod set at 2, 7, 7, 80, 10, 50, and 500, respectively.

### Analyses of rearranged chromosomes 4A, 5A, and 7B

The breakpoints that generated the rearrangements in wild emmer chromosomes 4A, 5A, and 7B were identified by examining the collinearity of the *Ae. tauschii* HC genes (queries) against genes annotated in the wild emmer pseudomolecules (targets) (Dvorak et al. 2018). In the reverse direction, 4A genes were used as queries against HC genes annotated in the *Ae. tauschii* pseudomolecules (targets) (Online Resource 1). Collinear genes on the query and target pseudomolecules that were closest to a switch in the target pseudomolecules delimited a region on the query pseudomolecule that harbored a breakpoint.

For each region harboring a breakpoint, collinear genes on the query pseudomolecule that were closest to the breakpoint on each side of it were recorded. A portion of the *Ae. tauschii* pseudomolecule including the pair of collinear genes was aligned against the BNG contigs on the four *Ae. tauschii* BNG maps as described below. Likewise, a portion of the wild emmer wheat pseudomolecule harboring the pair of genes flanking a breakpoint was aligned against the wild emmer wheat BNG contig. The correspondence of the Nt.*Bsp*QI restriction sites in the BNG contig and in the pseudomolecule sequence was visually examined for match and the presence of repeated structures.

In the analysis of the breakpoint located between 595.5 and 596.2 Mb on wild emmer wheat 4AL, a *T. aestivum* cv Chinese Spring (CS) BNG contig was aligned to the wild emmer BNG contig. The CS BNG contig was then used to align PacBio pseudomolecule 4A (http://aegilops.wheat.ucdavis.edu/ATGSP/dAetA.php) constructed from Pacific Bioscience (PacBio) long-read contigs (Zimin et al. 2017). Dot-plots were constructed for the CS pseudomolecule to determine the presence of satellite DNA.

### Sequence alignments on a BNG contig

The BNG map of wild emmer wheat Zavitan and four BNG maps of *Ae. tauschii* (Luo et al. 2017; Dvorak et al. 2018) were used. To compare a BNG contig with the nucleotide sequence of a pseudomolecule, the nucleotide sequence was digested in silico with the restriction endonuclease Nt.*Bsp*Q1 by using Knickers (BioNano Genomics). The alignment of a nucleotide sequence with the BNG contig or an alignment between BNG contigs was computed with RefAligner (BioNano Genomics). The alignment was visualized in IrysView (BioNano Genomics). Software packages for these operations were obtained from BioNano Genomics (https://bionanogenomics.com/support/software-downloads/).

## Results

### Global alignment

**Fig. 1:**
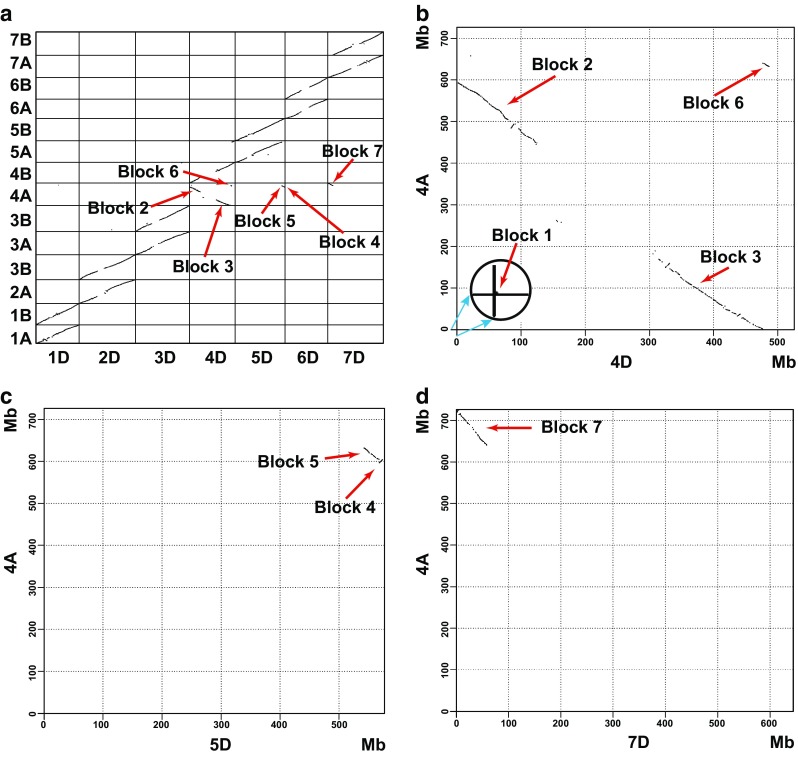
Dot-plots. **a** Dot-plots comparing the 14 wild emmer wheat pseudomolecules with the seven pseudomolecules of *Ae. tauschii*. Each dot consists of a sequence of three or more collinear genes. The plots are oriented with the tips of the short arms to the left (*x*-axis) and bottom (*y*-axis). Large gaps in the profiles are centromeric regions. The correspondence of alignments with the synteny blocks in Fig. 2a is indicated. **b**–**d** Details of synteny between wild emmer wheat pseudomolecule 4A and *Ae. tauschii* pseudomolecules 4D, 5D, and 7D, respectively. The correspondence of alignments with the synteny blocks in Fig. 2a is indicated in each figure. The antiparallel alignment (**b**) indicates a pericentric inversion starting with an interstitial breakpoint in present-day 4AL and ending with a breakpoint at the tip of the present-day 4AS delimiting block 1 (inset). The distal portion of 4AL consists of an inverted terminal portion of 5AL (including an additional distally located inversion) (blocks 4 and 5) (**c**), followed by a portion of 4AL (block 6) (**b**), and ending with an inverted terminal portion of 7BS (block 7) (**d**)

Dot-plots were constructed based on the global alignment of the wild emmer wheat A and B subgenomes against the *Ae. tauschii* pseudomolecules (Fig. 1a). The dot-plots confirmed that relative to pseudomolecule 4D, 4A was inverted along its entire length. The arm 4AS corresponded to 4DL. The proximal portion of 4AL corresponded to 4DS, which was then followed by a segment corresponding to the distal portion of 5DL, a small segment corresponding to 4DL, and ending with a segment that corresponded to the distal portion of 7DS. To summarize, starting at the tip of the present-day arm 4AS, the global dot-plots in Fig. 1a confirmed that the 4A pseudomolecule is homoeologous to the *Ae. tauschii* reference chromosome arms in the following sequence starting at the tip of the 4A short arm: 4DL-centromere-4DS-5DL-4DL-7DS.

**Fig. 2:**
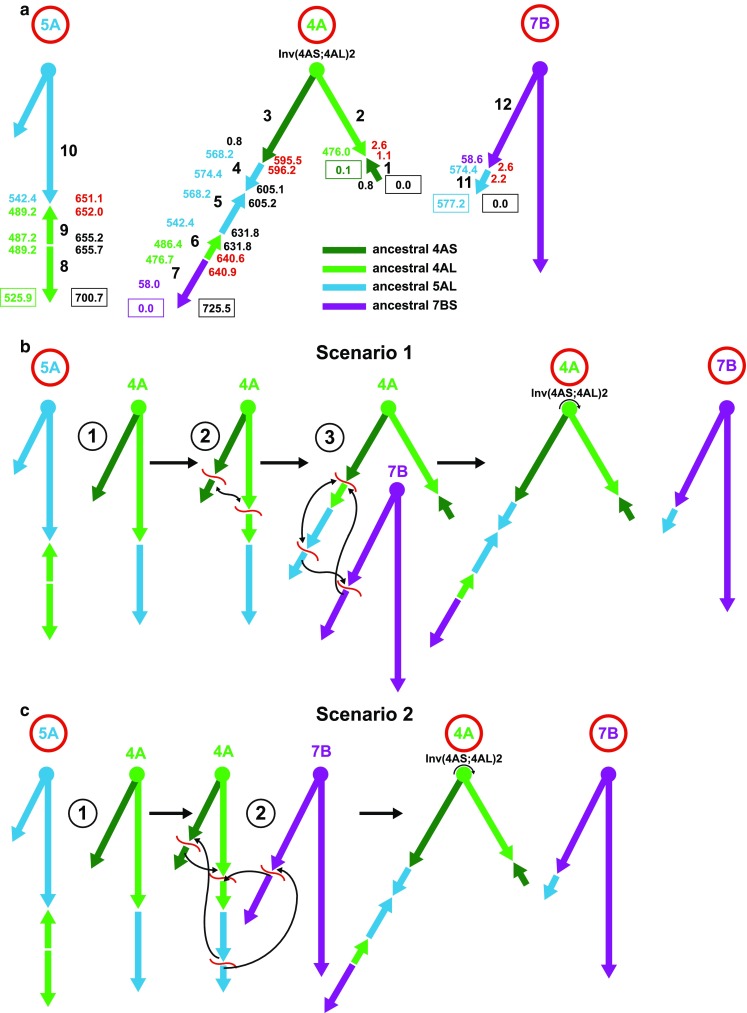
Evolution of wild emmer wheat 4A, 5A, and 7B reciprocal translocations and 4A inversions. **a** The structure of present-day chromosomes 4A, 5A, and 7B (indicated by red circles). The locations of major synteny blocks (black integers) in the *Ae. tauschii* pseudomolecules (coordinates are to the left of the chromosomes and are in green, dark green, blue, and magenta numbers corresponding to the ancestral chromosome or chromosome arm color designations) and their locations in the wild emmer pseudomolecules (coordinates are in black or red numbers to the right of the chromosomes). The colored arrows indicate the centromere-to-telomere direction of synteny blocks in the *Ae. tauschii* reference genome sequence. Centromeres are symbolized by ovals at the tops of inverted Vs. The main synteny blocks in wild emmer are numbered by large black integers from 1 to 12. The endpoint coordinates of pseudomolecules are boxed. The red numerals (Mb) to the right of the chromosomes indicate intervals spanning breakpoints. **b** Scenario 1 of step-wise evolution of reciprocal translocations involving chromosomes 4A, 5A, 7B and the pericentric and paracentric inversions in chromosome 4A. Numbers in black circles indicate steps in the evolution of the chromosomes. For the sake of clarity only the most relevant rearrangements are shown. The color coding is the same as in **a**. The three breaks at event 3, the paracentric inversion and T(4AL;7BS)1 are assumed to be simultaneous. **c** Scenario 2 in which the four breaks in step 2 resulting in the pericentric and paracentric inversions and the T(4AL;7BS)1 reciprocal translocation are assumed to have occurred simultaneously

To gain greater resolution, dot-plots were constructed for wild emmer wheat pseudomolecule 4A aligned against *Ae. tauschii* pseudomolecules 4D, 5D, and 7D (Fig. 1b–d). In agreement with the global alignment, the dot-plot involving 4D was inverted along its entire length confirming the existence of pericentric inversion Inv(4AS;4AL)1 spanning about 596 Mb of the 4A pseudomolecule (Fig. 1b). The plot showed a short synteny block (inset in Fig. 1b) homoeologous with the tip of 4DS (block 1 in Fig. 2a) that was expected to be at the tip of the present-day 4AS, based on the assumption that 4A was subjected to a pericentric inversion.

At about 596 Mb on the 4A pseudomolecule, the 4A pseudomolecule transited into a synteny block of about 40 Mb corresponding to the end of the *Ae. tauschii* arm 5DL (synteny blocks 4 and 5 in Figs. 1a, c, 2a). Synteny block 5 was in a reverse orientation relative to pseudomolecule 4D. Synteny block 4 was in the same orientation as it was in pseudomolecule 4D due to a paracentric inversion nested within paracentric inversion Inv(4AL;4AL)1. At about 640 Mb on the 4A pseudomolecule, there was a small synteny block of the ancestral arm 4AL (block 6 in Figs. 1a, b, 2a). The 4A pseudomolecule ended with block 7 (Figs. 1a, 1d, and 2a) corresponding to the tip of *Ae. tauschii* pseudomolecule 7DS. Block 7 was in an inverted orientation relative to the 7D pseudomolecule because the synteny block was in the long arm of 4A but in the short arm of 7D.

### Gene comparisons

To further increase resolution and locate the breakpoints of each rearrangement, we examined the spreadsheet matrix of BLASTP results using the 38,775 HC genes annotated in the *Ae. tauschii* pseudomolecules (Luo et al. 2017) as queries against the 65,012 HC genes annotated in the wild emmer pseudomolecules (Avni et al. 2017) used as targets (Dvorak et al. 2018). Similar homology searches were performed here, using the 4350 HC genes annotated in the wild emmer pseudomolecule 4A as BLASTP queries against the 38,775 HC genes annotated in the *Ae. tauschii* pseudomolecules used as targets (Online Resource 1). The top hits were arranged in a spreadsheet matrix as was done previously (Dvorak et al. 2018). The order of synteny blocks, their orientation, and the coordinates in Mb of genes flanking each breakpoint are graphically summarized for chromosomes 4A, 5A, and 7B in Fig. 2a.

### T(4AL;5AL)1

Since T(4AL;5AL)1 exists in diploid *T. monococcum* (Dubcovsky et al. 1996), the evolution of the wild emmer wheat chromosome 4A must have started with this translocation. Translocation T(4AL;5AL)1, explicitly named as TA(AET4Gv20754000:5100;AET5Gv21126000:200), interchanged a 4AL segment consisting of synteny blocks 8 and 9 with the 5AL synteny blocks 4 and 5 (Fig. 2a). The most parsimonious orientation of the ancient 4AL segment in 5AL was by the segment maintaining the original centromere-to-telomere polarity. However, synteny block 9 was inverted by inversion InvA(AET4Gv20755100-62600). This paracentric inversion shared the proximal breakpoint with the T(4AL;5AL)1 translocation breakpoint, which was between genes AET5Gv21126000 and AET5Gv21126200 on pseudomolecule 5D (Dvorak et al. 2018).

The ancestral 5AL segment translocated to the present-day chromosome arm 4AL was in an inverted orientation (Fig. 2a). The segment was subdivided into two synteny blocks 4 and 5. Synteny block 4 was inverted due to inversion InvA(AET5Gv21213200-35900). The inversion ended on its proximal side with the wild emmer gene TRIDC4AG047470 located at 596,507,146 bp on the 4A pseudomolecule. The proximal breakpoint of InvA(AET5Gv21213200-35900) was preceded by a block of three collinear genes TRIDC4AG047420 to TRIDC4AG047440, which were in the same orientation as synteny block 5 and represented the most distal portion of the translocated segment of the ancestral chromosome arm 5AL on the reference pseudomolecule 5D. The coordinates of these three genes on the 5D pseudomolecule indicated that the entire 5AL segment was originally in a reverse orientation, and inversion Inv(AET5Gv21213200-35900) was nested within the segment translocated from the ancestral 5AL arm.

### Inv(4AS;4AL)1

The pericentric inversion Inv(4AS;4AL)1 (event 2 in Fig. 2b), explicitly described as InvA(AET4Gv20002400:2700;AET4Gv20730000:100) based on the location of its breakpoints, took place in wild emmer and exchanged synteny block 1 with synteny blocks 4–6 (Fig. 2a). Synteny block 1 was detected by the 4A-4D dot-plot (Fig. 1b). It was short, 1,084,387 bp long and contained 9 collinear genes. The breakpoint between blocks 1 and 2 was detected in the present-day 4AS between wild emmer genes TRIDC4AG000110 (1,084,387 bp on the 4A pseudomolecule) and TRIDC4AG000170 (1,530,554 bp on the 4A pseudomolecule). An interval spanning the join was 446,167 bp long on the 4A pseudomolecule. The alignment of 4A against the 4D reference pseudomolecule suggested the existence of an intrachromosomal translocation involving five wild emmer wheat genes starting with TRIDC4AG000270 and ending with TRIDC4AG000350 translocated into synteny block 2. The translocated segment was not validated with the Zavitan BNG map and probably is an assembly error. The second breakpoint of Inv(4AS;4AL)1 was in a 324,864 bp interval (Online Resource 1) (Fig. 2a) between wild emmer genes TRIDC4AG047030 and TRIDC4AG047110.

### Inv(4AS;4AL)2

This inversion involves pericentromeric genes in the present-day 4AS and 4AL (Miftahudin et al. 2004; Ma et al. 2014). This inversion has been confirmed (Jorgensen et al. 2017), and it will not be dealt with here because it involves the pericentromeric region of 4A which we did not analyze.

### T(4AL;7BS)1

This translocation involved a 4A segment originally translocated from the ancestral 5A to 4A and a segment of the ancestral 7BS. Based on the locations of the breakpoints in the *Ae. tauschii* reference sequences, the translocation was explicitly named as TAB(AET5Gv21235900:39800;AET7Gv20264600:6900). The entire 7BS fragment was in synteny block 7. The block was in the opposite orientation to the progression of the 4A pseudomolecule, as indicated by the dot-plot (Fig. 1d), but it was in the same centromere-telomere orientation as it was in 7DS. Synteny block 7 was 81,791,655 bp long in the 4A pseudomolecule. The breakpoint of the T(4AL;7BS)1 translocation was between wild emmer wheat loci TRIDC4AG056470 at 640,594,166 bp and TRIDC4AG056510 at 640,877,889 bp (Online Resource 1). The interval between the two genes was 283,723 bp.

Using the *Ae. tauschii* 7D pseudomolecule as a reference, the breakpoint in 7B was located between AET7Gv20264500 (58,040,187 bp on the 7D pseudomolecule) and AET7Gv20266900 (58,660,865 bp on the 7D pseudomolecule). The fragment of 4AL(= 5AL) translocated to 7BS ended at *Ae. tauschii* locus AET5Gv21239800 and was only 2,222,006 bp, on the 5A pseudomolecule (block 11 in Fig. 2a). The polarity of block 11 was the same as in pseudomolecule 5D. A paracentric inversion spanned a region from 2,610,497 to 4,534,253 bp in the 7BS pseudomolecule. The inversion was not validated by the Zavitan BNG map and likely was an error in the wild emmer wheat assembly.

### Inv(4AL;4AL)1

This inversion, explicitly InvA(AET5Gv21235900-AET4Gv20730100), was proposed (Devos et al. 1995; Miftahudin et al. 2004) to account for the distal location of synteny block 6 relative to synteny blocks 5 and 4 and the inverted orientation of the three synteny blocks in the present-day 4AL. There should be breakpoints specific for this inversion on both sides of it, if the inversion happened as proposed.

The proximal side of the inversion included synteny block 4 (= 5AL). Synteny block 4 was inverted by paracentric inversion InvA(AET5Gv21213200-35900). However, that inversion was preceded by three genes TRIDC4AG047420 to TRIDC4AG047440 in pseudomolecule 4A already discussed in the context of T(4AL;5AL)1 (Online Resource 1). The genes were not involved in InvA(AET5Gv21213200-35900) suggesting that this inversion was nested within Inv(4AL;4AL)1. The most proximal gene in block 4 was TRIDC4AG047100 at 596,216,878 bp on the 4A pseudomolecule. This gene had an orthologue on the 5D pseudomolecule at 574,420,043 bp. The first 4A gene proximal to TRIDC4AG047100 with an orthologue in the 4D pseudomolecule was TRIDC4AG047030 (Online Resource 1). These two genes delimited a breakpoint region 383,209 bp long in the 4A pseudomolecule which could contain the Inv(4AL;4AL)1 proximal breakpoint. Importantly, there were no genes from blocks 5, 6, or 7 in this general area.

On the distal side of Inv(4AL;4AL)1 was synteny block 6 (ancestral 4AL), which was in an inverted orientation relative to the 4D reference sequence. It ended on the distal side with locus TRIDC4AG056470 (640,594,166 bp) that was orthologous to a 4D locus at position 476,689,202 bp on the 4D reference pseudomolecule. Neighboring locus TRIDC4AG056510 (640,877,889 bp on the 4A pseudomolecule) was on the 7D pseudomolecule and was the most proximal locus of synteny block 7. There was no 4D gene beyond TRIDC4AG056510, and no 5D gene located near this entire region, which is consistent with the breakpoint being in the 283,723 bp interval between TRIDC4AG056470 and TRIDC4AG056510.

### Nucleotide sequences at the breakpoint regions

To analyze the breakpoints at the nucleotide sequence level, the wild emmer wheat sequence scaffolds and the wild emmer wheat BNG contigs were aligned (Dvorak et al. 2018). Since *Ae. tauschii* pseudomolecules 4D, 5D, and 7D were used as references (Fig. 1b–d), the regions of the *Ae. tauschii* pseudomolecules corresponding to the breakpoints in the 4A pseudomolecule were aligned with *Ae. tauschii* BNG contigs to ascertain that no artefacts were present in the *Ae. tauschii* reference pseudomolecules (Online Resource 2). Except for a small discrepancy in a section of the 5D pseudomolecule, irrelevant to the problem at hand, the *Ae. tauschii* sequences at the breakpoints were validated by the BNG contigs.

**Fig. 3:**
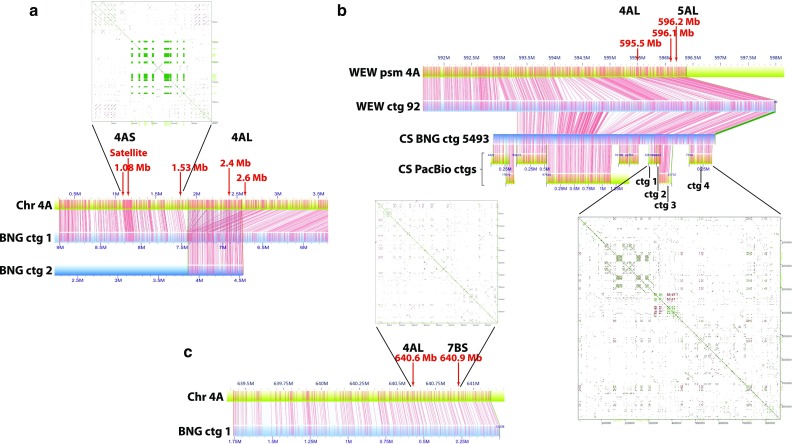
Details of the major breakpoints in chromosome 4A. Blue rectangles are BNG contigs and green rectangles are sequence scaffolds. All measures are in Mb. Red numbers provide locations in Mb of the collinear genes closest to breakpoints on the 4A pseudomolecule. **a** The breakpoint of the pericentric inversion joining syntenic blocks 1 and 2 in 4AS. The 1.9–2.6 Mb region was erroneously inserted during assembly as indicated by the correspondence of the 4A scaffold to two different BNG contigs. The actual breakpoint is between 1.08 and 1.53 Mb. The region contains satellite DNA (green section in the dot-plot in the inset). **b** The proximal breakpoint in 4AL joining ancient 4AS with ancient 5AL. The break is around or distal to 595.5 Mb. A region in the pseudomolecule distal to that point shows poor alignment to the corresponding BNG contig and appears to be misassembled in wild emmer wheat. The same region in the Chinese Spring pseudomolecule is correctly assembled, and it is contained in four separate sequence contigs. Contigs 1, 2, and 3 contain satellite DNA. **c** Distal break in 4AL joining an ancient 4AL segment (synteny block 6) with the 7BS segment (synteny block 7). The sequence aligned faithfully with the BNG contig. The pattern of restriction sites is not indicative of satellite DNA in that region. Except for a small region between 350,000 and 400,000 bp in the inset dot-plot, there is no evidence suggesting the presence of satellite DNA at this breakpoint

The alignment of the wild emmer pseudomolecule 4A and the Zavitan BNG contigs, including the Inv(4AS;4AL)1 breakpoint between TRIDC4AG000330 and TRIDC4AG000370 in present-day arm 4AS (1.08–2.6 Mb), revealed a misaligned segment at 2.04–2.46 Mb that aligned to Zavitan BNG contig 2 (Fig. 3a). Inspection of the sequence in that region revealed the presence of a short scaffold with a high number of Ns. Very likely, this scaffold was erroneously inserted within the breakpoint in the 4A pseudomolecule. However, the sequences at both boundaries of the breakpoint were assembled correctly (Fig. 3a). A region near the distal end of the breakpoint interval showed a high density of Nt.*Bsp*QI restriction sites, suggesting a repeated structure. A dot-plot of a 0.3 Mb interval (1.00–1.30 Mb on the 4A pseudomolecule) spanning this region revealed 8 regions containing satellite DNA (Fig. 3a). Since the regions formed an 8 × 8 square they must contain the same basic sequence. Further analyses of the satellite revealed that it consisted of 67 sequence motifs, most of them repeated several times. The motifs ranged in GC content from as little as 5% to as much as 81% (Online Resource 3).

The same analysis was performed with the other breakpoint of the Inv(4AS;4AL)1 pericentric inversion, which was located in the 665,863-bp region between wild emmer wheat genes TRIDC4AG047350 and TRIDC4AG047420 in the 4AL arm (Fig. 3b). This wild emmer wheat scaffold was also chimeric. While the proximal and distal portions of the breakpoint correctly aligned with BNG contig 92, the middle of the region did not (Fig. 3b). To obtain the missing sequence, *T. aestivum* cv CS pseudomolecule 4A (http://aegilops.wheat.ucdavis.edu/ATGSP/dAetA.php) constructed from Pacific Bioscience (PacBio) long-read contigs was aligned to the wild emmer wheat BNG contig at the breakpoint (Fig. 3b). There were four PacBio contigs at the breakpoint in the 4A pseudomolecule. The dot-plot revealed that PacBio contigs 2 and 3 near the 595.5 Mb boundary of the breakpoint also contained satellite DNA (Fig. 3b).

Finally, the sequence scaffold spanning the distal breakpoint of paracentric inversion Inv (4AL;4AL)1, between loci TRIDC4AG056470 and TRIDC4AG056510 and bridging the 4AL/7BS breakpoint (synteny blocks 6 and 7), and located at 640.6–640.9 Mb on the 4A pseudomolecule was analyzed (Fig. 3c). The 4A pseudomolecule aligned fully with the Zavitan BNG map. The distribution of the Nt.*Bsp*QI restriction sites did not indicate a repeated structure, which was confirmed by a dot-plot (Fig. 3c).

## Discussion

We identified a synteny block 1,084,387 bp long in the wild emmer wheat chromosome arm 4AS, which contained 9 HC genes that were collinear with *Ae. tauschii* genes located at the tip of arm 4DS. This synteny block was distal to synteny block 2 in wild emmer chromosome arm 4AS. Synteny block 2 was the remnant of the proximal portion of the ancestral chromosome arm 4AL, and genes in this block were collinear with genes in the long arm of *Ae. tauschii* reference pseudomolecule 4D. This is consistent with synteny block 2 being the remnant of the ancestral arm 4AL left in the present-day arm 4AS by pericentric inversion Inv(4AS;4AL)1. The 9 HC wild emmer wheat genes collinear with genes at the tip of 4DS were undoubtedly the synteny block 1, expected to exist if 4A was subjected to a major pericentric inversion (Devos et al. 1995; Mickelson-Young et al. 1995). Circo plots of the wild emmer wheat A and B subgenomes (Avni et al. 2017) show a single line connecting the tip of the 4BS pseudomolecule with the tip of the 4AS pseudomolecule, which is consistent with orthologous genes at the tips of the two homoeologous chromosome arms.

In agreement with a previous study (Jorgensen et al. 2017), the wild emmer wheat and *Ae. tauschii* reference genome sequence comparison failed to show evidence for breakpoints specific to the paracentric inversion Inv(4AL;4AL)1. The alignment of the 4A pseudomolecule on the *Ae. tauschii* reference sequence placed the proximal breakpoint of Inv(4AL;4AL)1 into an interval between loci TRIDC4AG047030 and TRIDC4AG047100. However, the same interval was shown to harbor one of the two breakpoints of the pericentric inversion Inv(4AS;4AL)1. On the distal side of Inv(4AL;4AL)1, a putative breakpoint of Inv(4AL;4AL)1 was placed into a 283,723 bp interval between loci TRIDC4AG056470 and TRIDC4AG056510. The same interval, however, harbored the breakpoint of the T(4AL;7BS)1 translocation.

There are two ways to account for the apparent colocation of the breakpoints of the paracentric inversion Inv(4AL;4AL)1 with the breakpoints of the pericentric inversion Inv(4AS;4AL)1 and reciprocal translocation T(4AL;7BS)1. One possibility is a recurrent breakage of chromosome 4A at the breakpoints of Inv(4AS;4AL)1 and T(4AL;7BS)1. A recurrent breakage could have produced Inv(4AL;4AL)1 without breakpoints specific for the inversion. Recurrent breaking of chromosomes has been inferred for mammalian and plant genomes (Murphy et al. 2005; Li et al. 2016; Dvorak et al. 2018). Recurrent breaking of 4AL between TRIDC4AG047420 and TRIDC4AG047350 and between TRIDC4AG056470 and TRIDC4AG056510 is one of the two assumptions that underlie scenario 1 accounting for the structure of present-day chromosomes 4A, 5A, and 7B (Fig. 2b). The other assumption of scenario 1 is that the three breaks in step 3 were simultaneous.

Both breakpoints of pericentric inversion Inv(4AS;4AL)1, one of which includes the proximal breakpoint of Inv(4AL;4AL)1, contain satellite DNA. Satellite DNA at the inversion breakpoint in the present-day arm 4AS consists of 67 motifs, some containing as much as 80% GC. Expansion of GC-rich repeats might lead to replication difficulties, fork stalling, and double-strand DNA breaks (DSB), which can lead to chromosome instability and rearrangements in heterochromatin (Peng and Karpen 2008). The presence of satellite DNA at the two breakpoints could be a factor in the recurrent breakage of 4A. About 6% of the breaks in the grass genomes break recurrently (Dvorak et al. 2018).

An alternative way to account for the absence of breakpoints specific for Inv(4AL;4AL)1 is to reject the sequential origin of the rearrangements and assume that all rearrangements happened simultaneously as indicated in Fig. 2c (step 2 in scenario 2). There are other scenarios possible to account for the structure of the wheat 4A, 5A, and 7B chromosomes, but they require additional assumptions. Because scenario 2 makes the least number of assumptions and postulates the fewest number of breakpoints in chromosomes 4A and 7B, four in scenario 2 compared to five in scenario 1, we prefer scenario 2 over scenario 1 in the evolution of wheat chromosomes 4A, 5A, and 7A.

## Author contribution statement

JD, M-CL, CMJ, TZ, LW, PEM, BSG, YQG, and AD planned the work. M-CL, KRD, and TZ performed BNG mapping and analyses; JD, LW, TZ, KMD, PQ, PEM, and YQG performed the analyses of genome structure and evolution. JD organized and managed the contributions to this publication and was primary author. All authors read and approved the final manuscript.

## Electronic supplementary material

Below is the link to the electronic supplementary material.

Supplementary material 1 (PDF 271 kb)

Supplementary material 2 (XLSX 259 kb)

Supplementary material 3 (XLSX 17 kb)
