# Supplementary material for: Reassessment of the evolution of wheat chromosomes 4A, 5A, and 7B
Source: Theor Appl Genet. 2018 Aug 23;131(11):2451–62. doi: 10.1007/s00122-018-3165-8 (PMC6208953; doi:10.1007/s00122-018-3165-8)
Supplement: Supplementary file 1 — Supplementary material 1 (PDF 271 kb) [file 122_2018_3165_MOESM1_ESM.pdf]

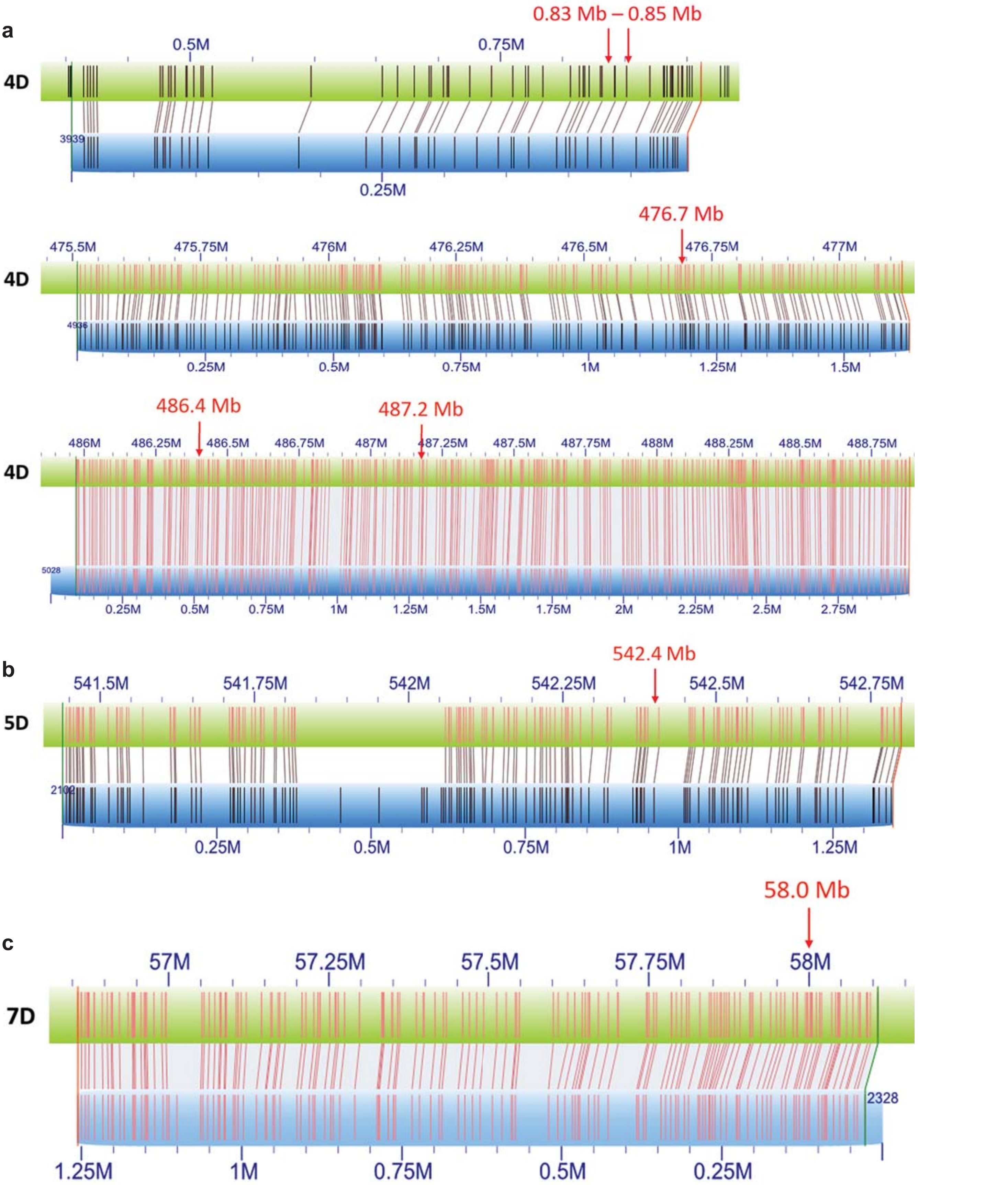

Fig. S1 Validation of *Ae. tauschii* genome sequence assembly in intervals corresponding to rearrangement breakpoints by aligning *Ae. tauschii* pseudomolecules (green rectangles) with the corresponding *Ae. tauschii* BNG contigs (blue rectangles). All measures are in Mb. Red numbers provide locations in Mb of breakpoints. **a** Comparisons of breakpoints involving chromosome 4D. The breakpoint between synteny blocks 1 and 2 are at top, the breakpoint distal to synteny block 6 is in the middle, and the breakpoint between synteny blocks 6 and 5 are at bottom. **b** Comparison of the breakpoint between synteny blocks 5 and 10 involving chromosome 5D. **c** Comparison of the rearrangement breakpoint between synteny blocks 7 and 12 involving chromosome 7D.
